# Staff quality of working life and turnover intentions in municipal nursing care and social welfare: a cross-sectional study

**DOI:** 10.1186/s12912-023-01339-0

**Published:** 2023-05-19

**Authors:** Maria Engström, Sofia Hanberger Jarnheden, Pia Tham

**Affiliations:** 1 Department of Caring Science, Faculty of Health and Occupational Studies, University of Gävle, Gävle, Sweden; 2 Medicine College, Lishui University, No. 1 Xueyuan Road, Lishui city, China; 3 Department of Social Work and Criminology, Faculty of Health and Occupational Studies, University of Gävle, Gävle, Sweden; 4 Department of Social Work, Uppsala University, Uppsala, Sweden

**Keywords:** Home-work interface, Intentions to leave, Job satisfaction, Nursing, Working life, Workload, Work-related stress

## Abstract

**Background:**

Nurses and social workers are two common professions with a university degree working within municipal nursing care and social welfare. Both groups have high turnover intention rates, and there is a need to better understand their quality of working life and turnover intentions in general and more specifically during the Covid-19 pandemic. This study investigated associations between working life, coping strategies and turnover intentions of staff with a university degree working within municipal care and social welfare during the Covid-19 pandemic.

**Methods:**

A cross-sectional design; 207 staff completed questionnaires and data were analyzed using multiple linear regression analyses.

**Results:**

Turnover intentions were common. For registered nurses 23% thought of leaving the workplace and 14% the profession ‘rather often’ and ‘very often/always’. The corresponding figures for social workers were 22% (workplace) and 22% (profession). Working life variables explained 34–36% of the variance in turnover intentions. Significant variables in the multiple linear regression models were work-related stress, home-work interface and job-career satisfaction (both for the outcome turnover intentions profession and workplace) and Covid-19 exposure/patients (turnover intentions profession). For the chosen coping strategies, ‘exercise’, ‘recreation and relaxation’ and ‘improving skills’, the results (associations with turnover) were non-significant. However, comparing the groups social workers reported that they used ‘recreation and relaxation’ more often than were reported by registered nurses.

**Conclusions:**

More work-related stress, worse home-work interface and less job-career satisfaction together with Covid-19 exposure/patients (Covid-19 only for turnover profession) increase turnover intentions. Recommendations are that managers should strive for better home-work interface and job-career satisfaction, monitor and counteract work-related stress to prevent turnover intentions.

## Introduction

Among both registered nurses and social workers turnover or turnover intentions have been quite common already pre-Covid-19 [1–4]. The Covid-19 pandemic has placed additional demands on healthcare staff globally [5], with high psychological distress for front-line workers [6], especially those in contact with Covid-19 patients [6–8]. Thus far, there has been a great deal of research on hospital staff (e.g., [5, 7, 9, 10] and less on staff working in municipal care and welfare [11, 12], e.g., registered nurses within elderly care and social workers within social welfare. Both groups have faced challenges and demands in their work due to the Covid-19 pandemic [13], but in different ways. Thus, the present study investigated working life and turnover intentions among staff with a university degree working in municipal care and social welfare, with a specific focus on registered nurses and social workers.

## Background

For registered nurses within elderly care, taking care of patients infected with Covid-19 and trying to prevent the transmission of Covid-19 in nursing homes have been challenging. High levels of job stress and fear for their own health have also been reported [11], along with low wellbeing [12]. Staff have reported stress related to the fear that they themselves, relatives and residents will be infected [12, 14]. Administrators have described the burden of unclear and contradictory guidelines [14], and staff have reported higher workload and emotional burden related to caring for infected residents, and residents being isolated due to visiting restrictions [14]. More home-work conflicts and decreased thriving have also been reported during Covid-19 as compared to pre-Covid measures [9]. In contrast, another study found levels of work-related quality of life similar to pre-pandemic levels [13], but levels had declined at the 12-moth follow-up [15]. Others have found that females [5, 7] and registered nurses report worse mental wellbeing compared to other healthcare staff in hospitals [7] and in nursing homes [11], and compared to physicians [5]. However, comparing different professions and countries, Lethin et al., [16] found that stress and anxiety levels among staff in elderly care differed across countries and professions, with the lowest levels among registered nurses in Sweden. Considering changes over time, an Australian study of physicians conducted during 2020 found improved mental wellbeing, less Covid-19 concerns and more support at the two-months follow-up [17]. Moreover, two additional studies conducted during 2020 found similar results, showing improved mental wellbeing at a short-term follow-up after the outbreak [18] and after a 6-month follow-up period [19]. Thus, the timing of the survey might also affect the results, emphasizing the importance of data collected at different time points as Covid-19 will be part of the society and something that we need to deal with for a long time.

For turnover intentions, an increase has been noted compared to pre-pandemic measures [2]. In a study among nurses, social workers and social care workers in elderly care during the Covid-19 pandemic, 51% reported turnover intentions in United Kingdom and 29% in Japan [20]. A Canadian study reported predictors such as leadership, fatigue, work satisfaction for intentions to leave both the profession and the workplace, whereas caring for Covid-19 patients, preparedness, and quality of care only predicted intentions to leave the profession [21]. They also found that registered nurses and licensed practical nurses caring for Covid-19 patients (46%) versus not reported more fatigue and lower work satisfaction [21] than those not caring for such patients. Another study using data collected in 2021 found that coronaphobia, coping and social support were related to the intention to leave both the organization and the profession [22]. A review of turnover intentions during Covid-19 found several individual and organizational factors related to turnover intentions including fear of Covid-19 and caring for Covid-19 patients [23].

For social workers, the demands of the Covid-19 pandemic entail the risk of increased vulnerability for their clients; individuals and families already experiencing socioeconomic disadvantages. For some marginalized families, the societal changes during the pandemic have meant additional hardships such as unemployment and isolation [24]. These strenuous conditions could increase the risk of further social suffering and exacerbated mental health needs within these groups for a long period. For some social workers, Covid-19 meant providing social interventions and support remotely, which impacted not only the way social workers engaged with clients, but also their connection with colleagues [25]. Remote work impacted the immediacy of peer support [26], which was particularly problematic for newer colleagues who had yet to form workplace relationships [25]. A systematic review, pre-pandemic, found that organizational culture was a crucial factor in turnover rates and that co-worker support acted as a buffer for burnout [27]. The extreme conditions during the pandemic likely meant that social workers were facing even greater health challenges. Nursing home social workers described feeling unprepared to meet the pandemic’s demands and challenges (53 of 63 with a degree in social work, 30 social service directors). They reported overwhelming stress, and increased workload [28].

In earlier studies, the use of strategies such as relaxation [13, 19, 20, 29, 30], resilience [7], exercise, [13, 19, 31], adequate knowledge, [7] and skill training during the Covid-19 pandemic has been linked to improved wellbeing and/or quality of working life [7, 13, 20, 29]. And education/training has been found related to turnover intentions [32]. With training, staff members’ self-efficacy in mastering work challenges can increase, and this, in turn, is related to greater wellbeing [7]. Thus, it was considered relevant to include these coping strategies in the present study.

### Theoretical framework

According to the Job-demand resource theory [33], high demands increase the risk of strain, and decrease wellbeing, whereas resources interact in a positive way increasing job engagement and decrease the negative impacts of demands. Examples of job demands are high workload and work-related stressors. In this connection, the Covid-19 pandemic has been one such stressor in both family and working life [9] and reported to be a common risk for decreased mental wellbeing [7, 8]. Job resources, for example job control, work-life balance and personal resources e.g., individual management/coping strategies can be used to reduce work strain [33]. In our study, the Job-demand resource theory is used as a framework for examining possible associations between working life, exposure to Covid-19, and turnover intentions.

To sum up, during the Covid-19 pandemic, a great deal of research has been done of hospital staff, whereas less research has focused on healthcare and social workers in municipal care and social welfare, especially regarding their intentions to leave the workplace or profession. Furthermore, a distinction between organizational and professional turnover intentions has been emphasized. Thus, the overall aim was to study associations between quality of working life, coping strategies and turnover intentions (profession and workplace) among all staff with a university degree working within municipal care and social welfare during the Covid-19 pandemic, and with a specific interest of registered nurses and social workers.

### Hypothesis 1

#### 1a

Access to job resources such as good ’home-work interface’ decrease staff members’ turnover intentions.

#### 1b

Access to job resources such as good control at work decrease staff members’ turnover intentions.

#### 1c

Access to job resources such as good working conditions decrease staff members’ turnover intentions.

#### 1d

Access to job resources such as high ’job-career satisfaction’ decrease staff members’ turnover intentions.

#### 1e

Few negative demands such as ’stress at work’ decrease staff members’ turnover intentions.

#### 1f

Few negative demands such as ’exposure of Covid-19’ decrease staff members’ turnover intentions.

### Hypothesis 2

#### 2a

Trying to cope with demands in working life during the Covid-19 pandemic using exercise is associated with lower rates of turnover intentions.

#### 2b

Trying to cope with demands in working life during the Covid-19 pandemic using ‘recreation and relaxation’ is associated with lower rates of turnover intentions.

#### 2c

Trying to cope with demands in working life during the Covid-19 pandemic such as improving skills is associated with lower rates of turnover intentions.

## Methods

### Design, setting and sample

The study had a cross-sectional correlational design. The setting was seven municipalities and a convenience sample of 626 participants with a university degree in healthcare or social work were invited from these seven municipalities. Inclusion criteria were (1) staff with a university degree in healthcare or social work and (2) working within municipal care and social welfare.

### Data collection

Data were collected during 2021, from March to September/October, using an online survey. Partly the same instruments as Mc Fadden et al., [13] and in collaboration with the research team from United Kingdom. Two reminders were sent to non-responders.

The *Work-related Quality of Life Scale* [34, 35] was used to assess working life. The 23-item scale consists of 6 factors: ‘general wellbeing,’ ’home-work interface,’ ‘job-career satisfaction,’ ‘control at work,’ ‘working conditions’ and ‘stress at work.’ These factors measure non-work (1 factor of general wellbeing, only used for total score of the *Work-related Quality of Life Scale*), work (4 factors all used in the present study as independent variables), and the link between work and non-work (1 factor of home-work interface, used in the present study as independent variable). Response alternatives range from (1) strongly disagree to 5) strongly agree. Factor scores are calculated by averaging the included item scores, and the total score is the average of all factor scores. Before calculating factor scores, negatively phrased items are reversed, for example the ones about stress at work, and thereby a high score of stress at work means less stress. Good construct validity and internal consistency have been reported; Cronbach’s alpha (α) ≥ 0.75 for all factors and total score. Higher scores indicate better work-related quality of life, i.e., that they more often agree with the statements/items in the factors ‘home-work interface,’ ‘job-career satisfaction,’ ‘control at work,’ ‘working conditions’ [34, 35]. To measure how staff mastered demands at work, we used three factors, each with three items, from the Work Stressor Coping Scale [36]: ‘working to improve skills/efficiency,’ ‘recreation and relaxation’ and ‘exercise.’ The response alternatives range from 1) never have done this to 6) always/almost always do this. The factor scores are the average of the included items’ scores. Good construct validity for the scale and good internal consistency, with α ≥ 0.76, for the factors have been reported [36]. The single-item measures about intention to leave [37] a) the profession or b) workplace were from earlier research [3, 4] developed for a study 2003 [37]; the response alternatives were on a 5-point scale; 1) never/very seldom, (2) rather seldom, (3) sometimes, (4) rather often, (5) very often/always [3, 4, 37]. Furthermore, participant demographic data were collected, including exposed to Covid-19 infection (whether they had had it themselves, relative(s), whether they had seen patients/clients who have had it and/or whether they had cared for patients/clients with an ongoing Covid-19 infection, study specific question).

### Data analysis

Data were analyzed using IBM SPSS Statistics 27, Spearman’s rho (*r*_s_) and multiple linear regression models. In line with job-demand resource theory, we regarded exposure to Covid-19 and ‘stress at work’ as demands, and ’home-work interface,’ ‘job-career satisfaction,’ ‘control at work,’ ‘working conditions,’ ‘working to improve skills/efficiency,’ ‘recreation and relaxation’ and ‘exercise’ as resources. Variables with p-values ≤ 0.10 in the univariate and bivariate analyses were included as independent variables in the multiple regression models (i.e., ’home-work interface,’ ‘job-career satisfaction,’ ‘control at work,’ ‘working conditions, ‘stress at work’ and, exposure to Covid-19). Age was controlled for in all models. The residuals from the models were visually inspected with histograms and Q-Q plots and showed no serious deviation from a normal distribution. Variance inflation factor values were checked, and all values were below 2.1, indicating no multicollinearity problems. To test regression there is a recommendation of N ≥ 50 + 8 times the number of independent variables [38]. Thus, with seven independent variables as in our study the sample size should be at least 106 (50 + [8*7]). The significance level for the analyses was p ≤ .05.

### Ethical considerations

The study was approved by the Swedish Ethical Review Authority (Reg. no. 2020–05487). All participants received written information about the study, voluntary participation and were assured confidentiality.

## Results

Two hundred and seven staff responded to the questionnaire (response rate 33%), most were female (90%), and worked as frontline registered nurses in elderly care (32%) or social workers (53%); their mean age was 46 years. The participants had worked an average of around 12.5 years in their respective professions. For participant characteristics see Table 1.

**Table 1:** Participants’ characteristics

Age, years, mean (SD), n = 206	46.0 (10.9)
Years worked in the profession, mean (SD), n = 205	12.5 (10.3)
Years worked, this employer, mean (SD), n = 207	9.6 (9.8)
Women, n (%)	186 (89.9%)
*Exposure Covid-19 yes infected* n (%)	All n = 185
- myself	55 (29.7%)
- relatives(s)	92 (49.7%)
- patient(s)/client(s)	136 (73.5%)
*Profession n (%)* n = 207	
Registered Nurses elderly care (18 with a specialist degree in nursing)	67 (32.4%)
Social Workers	109 (52.7%)
Occupational therapists	7 (3.4%)
Physiotherapists	5 (2.4%)
Managers	13 (6.3%)
Others (e.g., care coordinator)	6 (2.8%)

Abbreviations: SD standard deviation

### Turnover intentions

Among all staff 22% thought of leaving the workplace and 19% the profession ‘rather often’ and ‘very often/always’. The corresponding figures for social workers were 22% (unit) and 22% (profession) and for registered nurses 23% (unit) and 14% (profession) (Table 2). Regarding infection with Covid-19, 29.7% of the staff had been infected with Covid-19, 49.7% reported that their relative(s) had been infected, 73.5% had seen patient(s)/client(s) that had been infected by Covid-19, and 89% of the registered nurses had cared for patient(s) with an ongoing Covid-19 infection. Comparing turnover intentions of those responding yes or no for (a) infected myself, (b) relative(s) and (c) had seen patient(s)/client(s) that had been infected significant differences were found for ‘had seen patients/clients…’ (turnover intentions profession p 0.048, whereas for workplace the p-value was 0.076 [Mann-Whitney U test, 2-sided test]).

**Table 2:** Turnover intentions, profession and workplace

	n (%)					*Mean (SD)* *p value*
*Intention to leave profession*	*Never/Very seldom*	*Rather seldom*	*Sometimes*	*Rather often*	*Very often/ Always*	
All	76 (37.3%)	34 (16.7%)	56 (27.5%)	26 (12.7%)	12 (5.9%)	2.3 (1.2)
Social workers	33 (30.3%)	20 (18.3%)	32 (29.4%)	18 (16.5%)	6 (5.5%)	2.5 (1.2) p = .145^†^
Registered nurses	28 (43.1%)	8 (12.3%)	20 (30.8%)	4 (6.2%)	5 (7.7%)	2.2 (1.3)
… workplace						
All	67 (32.8%)	38 (18.6%)	55 (27.0%)	27 (13.2%)	17 (8.3%)	2.5 (1.3)
Social workers	33 (30.6%)	21 (19.4%)	30 (27.8%)	14 (13.0%)	10 (9.3%)	2.5 (1.3) (p = .846^†^)
Registered nurses	20 (30.8%)	10 (15.4%)	20 (30.8%)	10 (15.4%)	5 (7.7%)	2.5 (1.3)

^†^ Mann Whitney U test comparing social workers and registered nurses. When count does not add to 207 for all, 109 for social workers and 67 for registered nurses there are internal missing values.

### Associations between quality of working life, coping strategies and turnover intentions

In the bivariate analyses, statistically significant positive associations were found for the outcomes (turnover intentions profession or workplace) and the independent variables ‘home-work interface,’ ‘job-career satisfaction,’ ‘control at work,’ ‘working conditions,’ ‘stress at work’ (high values = less stress),’ age (except for turnover intention workplace) (Table 3). The mean value for total score of work-related quality of working life was 3.7 and standard deviation 0.5, minimum 2.0 and maximum 5.0 (the possible range for the scale is 1 to 5).

None of the coping strategies were statistically significant associated with turnover intentions and thus not included in the multiple linear regression models. Regarding coping strategies, the scores for social worker were ‘recreation relaxation’ mean 3.7 (SD 0.9), ‘exercise’ 3.6 (1.1) and ‘improving skills’ 4.1 (0.8). For registered nurses the scores were ‘recreation relaxation’ mean 3.3 (SD 1.0), ‘exercise’ 3.5 (1.2) and ‘improving skills’ 4.0 (0.9). The response alternatives for the items in the factors ranged from 1) never have done this to 6) always/almost always do this. Comparing registered nurses and social workers, results showed statistically significant higher score i.e., more use of ‘recreation relaxation’ among social workers than registered nurses (Mann-Whitney U test, 2-sided test, p = .007). For the other coping strategies, the results were non-significant.

**Table 3:** Bivariate associations between study variables, Spearman’s rho (p-value)

Quality of working life	Intention to leave profession	Intention to leave workplace	Cronbach’s Alpha
Home-work interface	− 0.337 **(≤ 0.001)**	− 0.347 **(≤ 0.001)**	0.74
Job-career satisfaction	− 0.415 **(≤ 0.001)**	− 0.462 **(≤ 0.001)**	0.72
Control at work	− 0.238 **(0.001)**	− 0.307 **(≤ 0.001)**	0.64
Working conditions	− 0.385 **(≤ 0.001)**	− 0.350 **(≤ 0.001)**	0.67
Stress at work	− 0.305 **(≤ 0.001)**	− 0.329 **(≤ 0.001)**	0.85
**Coping strategies**			
Recreation Relaxation	0.028 (0.691)	0.003 (0.968)	0.73
Exercise	0.048 (0.491)	0.008 (0.908)	0.74
Improve skills	− 0.075 (0.289)	− 0.097 (0.169)	0.74
Age	− 0.215 **(0.002)**	− 0.137 (0.051)	

Boldface type indicates statistically significant values.

In the multiple linear regression models, the significant variables for both turnover intention profession and workplace were home-work interface, job-career satisfaction, stress at work, and age, whereas ‘have seen patients/clients with Covid-19’ was only significant for turnover intentions profession. The negative associations indicate that higher scores i.e., better home-work interface, higher job-career satisfaction, stress at work (high scores = less stress), and older age are related to lower rates of turnover intention. The models explained 36% of the variance in turnover intentions profession and 34% of the variance in turnover intentions workplace (Table 4).

**Table 4:** Multiple linear regression analyses with the outcome variables turnover intentions profession and workplace

	Turnover intentions profession n = 178	Turnover intentions workplace n = 179
	Standardized beta coefficients (p-value)	Standardized beta coefficients (p-value)
Home-work interface	− 0.240 **(0.005)**	− 0.180 **(0.035)**
Job-career satisfaction	− 0.250 **(0.005)**	− 0.304 **(0.001)**
Control at work	0.055 (0.492)	− 0.047 (0.563)
Working conditions	0.002 (0.983)	0.055 (0.539)
Stress at work	− 0.269 **(≤ 0.001)**	− 0.267 **(≤ 0.001)**
Age	− 0.235 **(≤ 0.001**)	− 0.158 **(0.018)**
Covid-19, met patients/clients, no 1, yes 2	0.126 **(0.048)**	0.105 (0.103)
R2/R2 adjusted	0.36/0.33	0.34/0.31

Boldface type indicates statistically significant values.

Thereby, Hypotheses 1a-f were confirmed in the bivariate analyses (Table 3), except for the job demand ‘exposure to Covid-19 infection’ for turnover intentions workplace (1f); and in the multiple regression models significant variables were home-work interface, job-career satisfaction, stress at work and Covid-19 exposure (Table 4). Hypotheses 2a-c, about coping strategies, were not confirmed (Table 3).

## Discussion

The study objective was to study associations between quality of working life, coping strategies and turnover intentions (profession and workplace). The significant factors that increased turnover intentions in the models were contact with Covid-19 patients/clients (only for leaving the profession), less job-career satisfaction, together with worse home-work interface, more work stress and younger age. This is similar to results from a Canadian study [21], which found that work satisfaction was a predictor of both kinds of turnover intentions and that caring for Covid-19 patients was a predictor of intentions to leave the profession. The results are in line with the JD-R theory [33] and similar as in a review of nurses’ turnover intentions during Covid-19 [23] and in a review of turnover and turnover intentions before Covid-19 e.g., stress and job satisfaction [39]. In an interview study of factor that may push nurses to leave profession limited career opportunities (cf. our job-career satisfaction) were mentioned and they suggested more family-friendly roster (cf. our home-work interface) [40]. A review [41] of factors related to actual turnover of newly registered nurses found for example intention to stay, job satisfaction, older age, physical and emotional exhaustion, and worse health status as positively or negatively related factors. In our study, using the coping strategies, ‘exercise’, ‘recreation & relaxation’, and ‘improving skills’ were not associated with lower rates of turnover intentions neither in the bivariate nor the multiple regression analyses. Others have found being prepared [21] and education/training important for turnover intentions during Covid-19 [32] and ‘recreation and relaxation’, ‘improving skills’ [13, 42] and ‘exercise’ [42] related to quality of working life.

Our results on quality of working life (mean score 3.7) were slightly higher/better than reported in the study from United Kingdom (mean = 3.4) [13], and among healthcare workers pre-pandemic (mean = 3.4) [34]. Several studies have reported worse mental health during Covid-19 [8, 11]. However, our data were collected in 2021, and it may be that staff had become more used to Covid-19, had improved their knowledge and Covid-19 vaccination had occurred, which may have led to less Covid-19-related stress. Prospective studies during 2020 also showed improved wellbeing/decreased distress 1-2-months after the outbreak [17, 18] and at a 6-month follow-up [19], whereas others [15, 42] found the opposite for both wellbeing and quality of working life. Interviews with nurses caring for Covid-19 patients [43] showed similar results with a peak in negative emotions during the early stage of the outbreak and more positive emotions gradually appearing along with different adjustments. However, in contrast to our study, an Irish study of nursing home staff [11] conducted from November 2020 to January 2021 found low wellbeing, with the lowest scores observed among registered nurses compared to others. In their study, most staff (64%) had not cared for patients with Covid-19, whereas in our study, 89% of the registered nurses had cared for patients with Covid-19 and among all staff 74% had met patients/clients who had been infected. One study [10] found greater wellbeing among those who had taken care of more infected patients. Though, a review study of research conducted during the first year of the Covid-19 pandemic [7] found contact with Covid-19 infected patients as a risk for poorer mental health. Thus, the results might differ depending on when data have been collected during the Covid-19 pandemic and depending on the peaks in different countries.

For registered nurses 23% thought of leaving the workplace and 14% the profession ‘rather often’ and ‘very often/always’ (for all staff the corresponding figures were 22% and 19%). Others have found similar results for registered nurses [32] but also higher percentage of turnover intentions in samples of nurses or mixed professions [2, 20, 21]. The high rates of turnover intentions are a discouraging results, as it indicates Covid-19 as a risk that more staff may leave the profession. The scores for use of the coping strategies ‘exercise’, ‘recreation and relaxation’, and ‘improving skills’ in our study were similar as reported by Gillen et al., [15].

### Clinical implications

Our results highlight the need of better home-work interface, job-career satisfaction, and less work-related stress to prevent turnover intentions. With regards to home-work interface the manager and co-workers need to discuss this topic to find suitable adjustable solutions for the staff. Work-related stress needs to be discussed and monitored in order to counteract it. Furthermore, staff sense of thriving (i.e., both a sense of and satisfaction with learning and vitality) and access to empowering structures such as resources, support, and opportunities to professional growth needs to be monitored and discussed within the group together with the manager as thriving and resources have been found related to turnover [1] and empowering structures to job satisfaction [44, 45]. Managers striving for better home-work interface for their staff, job-career satisfaction, and less work-related stress are important to decreased turnover intentions among staff.

### Limitations

Limitations are the cross-sectional design, which limits conclusions about causal relationships, the convenience sample and low response rate, which limit generalizability. Furthermore, the use of self-reported data always has the risk of bias such as social desirability. Strengths of the study are that valid and reliable instruments were used, α levels present study > 0.70 for all study variables except two factors (control at work 0.64 and working conditions 0.67). Recommendations for future studies are the use of longitudinal design to study turnover intentions over an extended period and also actual turnover.

## Conclusions

Resources decreasing the risk of turnover intentions are better ‘home-work interface’ and ‘job-career satisfaction’ and demands increasing the risk are work stress, and Covid-19 (Covid-19 only for intention to leave the profession).

## Data Availability

The summary data are in the main document. Research data (the data set with individual data) are not shared. Individual data are not available due to general data protection regulations (GDPR), and in line with the ethics application. Aggregated data related to study results are available from the corresponding author upon reasonable request.

## Abbreviations

α: Cronbach’s alpha
Covid-19: Coronavirus Disease 2019
